# Convergence and translation: attitudes to inter-professional learning and teaching of creative problem-solving among medical and engineering students and staff

**DOI:** 10.1186/1472-6920-14-14

**Published:** 2014-01-22

**Authors:** Howard Spoelstra, Slavi Stoyanov, Louise Burgoyne, Deirdre Bennett, Catherine Sweeney, Hendrik Drachsler, Katrien Vanderperren, Sabine Van Huffel, John McSweeney, George Shorten, Siun O’Flynn, Padraig Cantillon-Murphy, Colm O’Tuathaigh

**Affiliations:** 1 Welten Institute, Research Centre for Learning, Teaching and Technology, Open University of the Netherlands, Valkenburgerweg 177, 6419 AT Heerlen, Netherlands; 2 School of Medicine, Brookfield Health Sciences Complex, University College Cork, Cork, Ireland; 3 Department of Electrical Engineering, ESAT-SCD, and iMinds Future Health Department Katholieke Universiteit Leuven, B-3001 Leuven, Belgium; 4 Department of Electrical and Electronic Engineering, University College Cork, College Road, Cork, Ireland

**Keywords:** Translation, Interdisciplinary learning, Creativity, Medicine, Engineering

## Abstract

**Background:**

Healthcare worldwide needs translation of basic ideas from engineering into the clinic. Consequently, there is increasing demand for graduates equipped with the knowledge and skills to apply interdisciplinary medicine/engineering approaches to the development of novel solutions for healthcare. The literature provides little guidance regarding barriers to, and facilitators of, effective interdisciplinary learning for engineering and medical students in a team-based project context.

**Methods:**

A quantitative survey was distributed to engineering and medical students and staff in two universities, one in Ireland and one in Belgium, to chart knowledge and practice in interdisciplinary learning and teaching, and of the teaching of innovation.

**Results:**

We report important differences for staff and students between the disciplines regarding attitudes towards, and perceptions of, the relevance of interdisciplinary learning opportunities, and the role of creativity and innovation. There was agreement across groups concerning preferred learning, instructional styles, and module content. Medical students showed greater resistance to the use of structured creativity tools and interdisciplinary teams.

**Conclusions:**

The results of this international survey will help to define the optimal learning conditions under which undergraduate engineering and medicine students can learn to consider the diverse factors which determine the success or failure of a healthcare engineering solution.

## Background

Increasingly, physicians are expected to operate effectively within a holistic, multi-disciplinary model of healthcare delivery. The UK General Medical Council (GMC) requires that medical schools provide formal curricular interdisciplinary learning opportunities to undergraduate medical students [1]. The benefits of interdisciplinary practice across a variety of healthcare contexts are well documented; poor relations and communication between physicians and allied health professionals contributes to decreased patient satisfaction and poorer overall treatment outcomes [2-4].

Expertise in engineering and medicine/healthcare is prerequisite to the design of biomedical devices and healthcare solutions. However, the benefits of a multidisciplinary approach to the design of medical devices, for example, exceed simply access to one standard knowledge base. Innovators with medical and engineering backgrounds may bring scientific method (characterized by experiment and requirement for repeatability) and experience of design process (fuzzy and non-linear) in differing degrees. This dual perspective and methodology should (and has) enhance medical device development [5]. The dividend proposed for such an intensive and challenging approach (multidisciplinarity) is user-readiness and minimization of obstacles to implementation. There is a recognised need for graduates equipped with the knowledge and skills to apply interdisciplinary medicine/engineering approaches to the development of novel healthcare solutions [5]. Recently, commentators have emphasised the importance of introducing innovation and creativity training into undergraduate medical curricula as a means of enhancing scientific innovation in medicine [6].

Project-based undergraduate modules have been described in which medical and engineering students work in multidisciplinary teams to develop solutions to a diverse set of health challenges, using a bioengineering design model [7,8]. The process can, however, be confounded by differences between doctors and engineers, notably the tendency of doctors to privilege a biomedical, and engineers a technical perspective to a given clinical design problem [9]. Other obstacles include the use of brain-storming-type, unstructured, innovation activities which are largely *ad hoc* and reliant on luck [10]. These illustrate the importance of bridging this innovation-impairing gap at an undergraduate level, in order to produce more effective medical and engineering innovators for the future [11].

As part of a multinational EU-funded project, BioApp [http://www.bioapp.eu], we have recently described the establishment of biomedical design module which brings together medical and engineering students at the senior undergraduate level [12]. Student teams, each with members of both disciplines, select one real-world clinical problem from a menu of options, and are paired with a senior clinician who acts as clinical mentor over a 12 week semester. The teams receive instruction on the use of the structured creativity tool *TRIZ* (translated as the’theory of inventive problem solving) [13], a multi-stage ideation process which enables students to develop novel and innovative design solutions. The learning objectives associated with this module are (a) to enhance co-operation in a multidisciplinary team activity, (b) to facilitate participation in a conceptual design process including proposals and justification of a value proposition, (c) to enable the conduct of multidisciplinary analysis of real-world clinical problems, (d) to enable students to present analysis to stakeholders from different backgrounds. In many of the existing interdisciplinary modules, the primary focus is on the project output, with little considerations of the barriers and facilitators to effective interdisciplinary learning; in this module, however, the focus is on the pedagogical process.

In order to fine-tune the module design and assess the factors which may promote/hinder effective interdisciplinary teamwork, we conducted a systematic survey of (i) current gaps and deficiencies in the needs, knowledge and practice associated with interdisciplinary learning and teaching, and (ii) promotion of innovation and creativity, in engineering and medical schools in two EU countries.

## Methods

### Study design

We used a quantitative questionnaire-based approach to chart knowledge and practice in interdisciplinary learning and teaching, and teaching of innovation, in engineering and medical schools in two European universities. Comparison of undergraduate medical and engineering curricula *across the selected institutions* indicated a high degree of alignment with respect to content coverage, exit outcomes, as well as interprofessional/interdisciplinary activities. Development of questionnaire content and format involved: (a) a review of legacy systems *via* consultation with the key stakeholders across academic engineering [biomedical, civil, electrical and electronic, mechanical] and medical departments in the Katholieke Universiteit Leuven [KU-L; Belgium] and University College Cork [UCC; Ireland]; (b) consultation with an expert panel composed of medical and engineering colleagues from within the wider partner consortium [KU-L, UCC, Open Universiteit Nederland] who are working directly in this field. This study was approved by the Clinical Research Ethics Committee of the Cork Teaching Hospitals.

The 25-item questionnaire was designed to examine various domains relevant to knowledge and practice in interdisciplinary learning and teaching, as well as how creativity and innovation are taught in engineering and medical schools. Staff and student versions of the questionnaires were designed with overlapping question content across both versions allowing between-group comparisons on the same dimension. The staff questionnaire contained the following categories: demographics, general attitudes towards interdisciplinary learning and creativity, personal pedagogical style, organisational issues, content, instructional design, and assessment. The student version contained the same categories, except the ‘content’ category. The response format consisted of binary items, ranking-based items, several open-ended questions, and a series of statements accompanied by Likert-scale options [on a scale of 1–5 from strongly disagree to strongly agree]. The complete questionnaire is available at the following web-address: http://www.bioapp.eu/1grfwfff4ad?a=1&p=21523375&t=20484775.

A web-based invitation to complete the questionnaire (hosted by Questback, http://www.questback.com) was distributed via e-mail to all staff and students across medical and engineering schools at KU-L and UCC. The web-based surveys were open from 03.02.2012 until 11.03.2012.

### Data analysis

Summary statistics (means [M] and standard deviation [SD]) were used to summarise Likert-scale responses for staff and students; Mann–Whitney U tests were used to measure differences between both groups (medical *vs*. engineering students/staff) where the outcome variable(s) consisted of Likert-scale question responses. Statistical analyses were carried out using PASW Statistics 18 [IBM, New York, NY, USA].

## Results

A total of 159 staff and 633 student members responded. The response rate across the schools was as follows: *staff* – engineering (25.96%), medicine (25.48%); *students* - engineering (17.10%), medicine (29.31%).

### Staff

The gender distribution of staff member respondents was: female (25.8%), male (74.2%). All staff respondents worked in the engineering (66.7%) or medical school (31.4%), or some other faculty (2.5%). Their educational backgrounds varied from medical (23.3%) to engineering (61.6%) or both medical and engineering (6.3%). Nine per cent of the sample claimed to have some other academic background. Age stratification, in years, was as follows: 20–29 (37.7%); 30–39 (18.9%); 40–49 (19.5%); 50–59 (17.6%); 60 and over (6.3%).

### Students

The gender distribution of student respondents was as follows: female (42.0%), male (58.0%). They were based in the engineering (48.2%) or medical school (51.8%), or other faculty (0.8%). Their reported academic and research interests spanned the medical field (34.4%), engineering field (23.7%), or both (41.9%). The age profile of student respondents, in years, ranged from 17 to 48, with a median age of 21.

### General attitudes towards interdisciplinary learning

**Figure 1:**
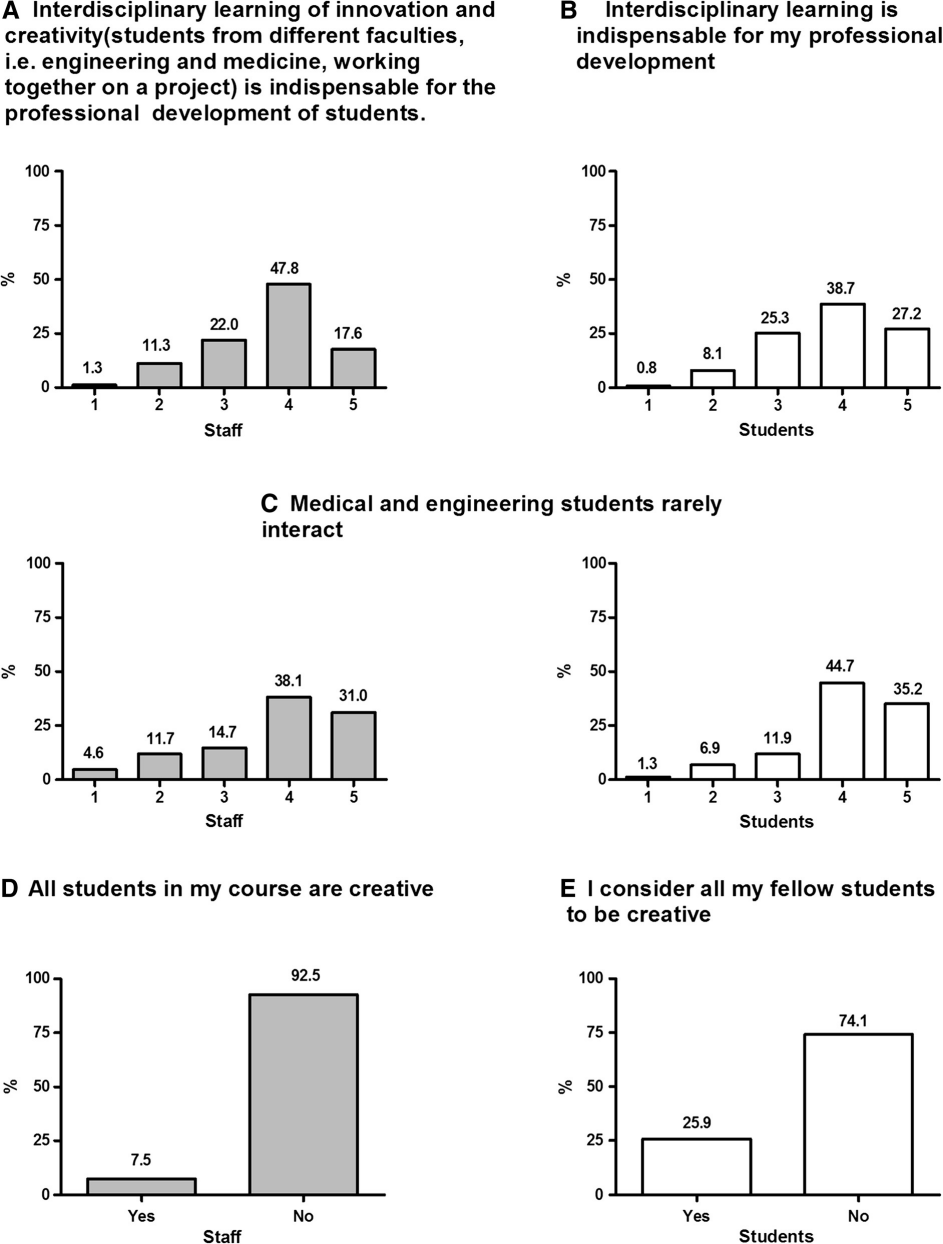
**Total percentage values for staff and students across agreement ratings [1–5, from strongly disagree to strongly agree] for statements: A-B**. Interdisciplinary learning is indispensible for professional development of students. **C**. Medical and engineering students rarely interact. **D & E**: Total percentage of Yes *vs*. No responses for staff and students for statement ‘All students in my course are creative’.

Both staff (M = 3.69; SD = 0.93) and students (M = 3.83; SD = 0.94) surveyed supported the idea that interdisciplinary learning is necessary for professional development of students (see Figures 1A & B). Both staff and students agreed that medicine and engineering students rarely interact in an academic setting (M _staff_ = 4.06, SD = 0.93; M _students_ = 3.79, SD = 1.40; see Figure 1C), and that they used different vocabularies (M _staff_ = 4.13, SD = 0.79; M _students_ = 3.70, SD = 0.92). Students expressed a weak preference for not working and learning in interdisciplinary groups (M = 2.89; SD = 0.90), preferring to work and learn with their course colleagues. With respect to creativity, a very low percentage of the staff (7.5% ‘Yes’) and students (26% ‘Yes’) agreed with the statement that ‘every student is creative’ (see Figures 1D & E).

Most staff (82.4%) and students (71%) do *not* believe that creativity is an unchangeable personality trait. However, opinions were divided among staff (59.7% ‘Yes’) and students (52.6% ‘Yes’) whether creativity can be learned. Both staff and students strongly disagreed with the statement that providing methods for problem solving hinder creativity (‘No’ response: 91% of staff *vs*. 80.4% of students).

### Personal teaching and learning styles

**Figure 2:**
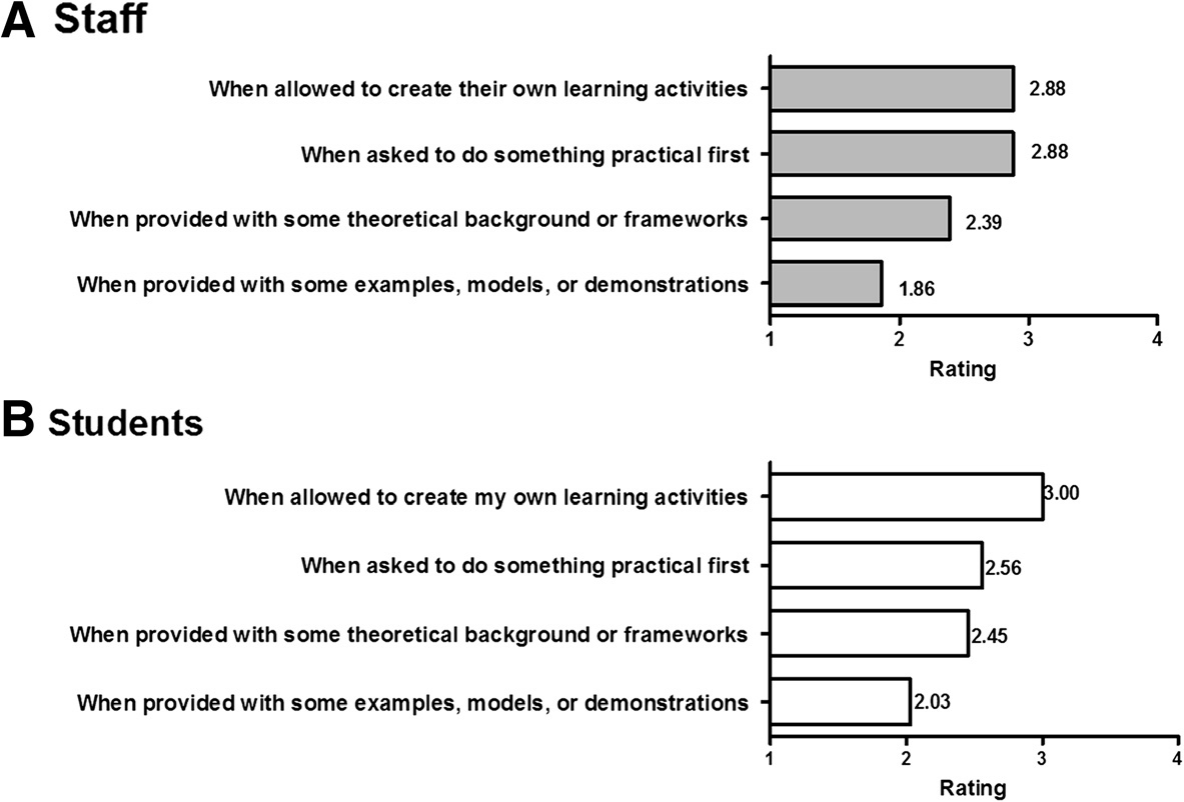
Mean rankings for students’ learning styles [A] and staff teaching styles [B] on a scale of 1–4, where each of the styles are ranked according to preference.

The questionnaire referred to the personal teaching and learning styles as categorized by Honey and Mumford (reflector, theorist, activist, and learning locus of control) [14]. Reflector and theorist styles were the least preferred styles of teaching for staff (M _reflector_ = 1.86, SD = 1.00; M _theorist_ = 2.39, SD = 1.10) and learning for students (M _reflector_ = 2.00, SD = 1.08; M _theorist_ = 2.45, SD = 1.06). The learning locus of control style (creating one’s own learning activities) obtained the greatest ranking score from both staff (M = 2.88, SD = 1.02) and students (M = 3.00, SD = 1.08) (see Figures 2A & B).

### Organisational issues

**Figure 3:**
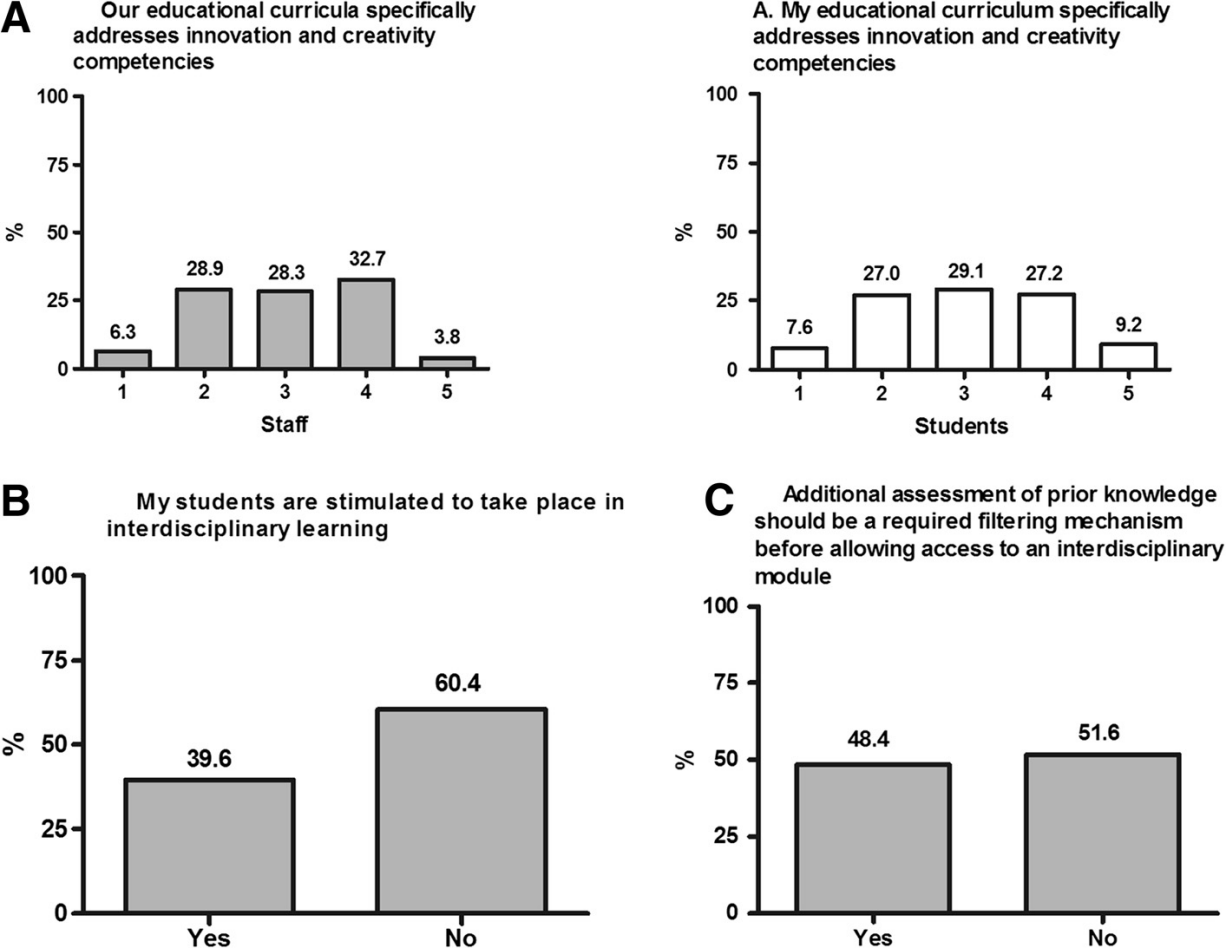
**Staff and student agreement ratings for organizational issues. A**. Total percentage values for staff and students across agreement ratings [1–5, from strongly disagree to strongly agree] for statement ‘Our educational curricula specifically addresses creativity and innovation’. **B**: Total percentage of Yes *vs*. No responses from staff for statement ‘My students are stimulated to take place in interdisciplinary learning’. **C**: Total percentage of Yes *vs*. No responses from staff for statement ‘Additional assessment of prior knowledge should be a required filtering mechanism before allowing access to an interdisciplinary module’.

Neither staff (M = 2.99; SD = 1.00) nor students (M = 3.03; SD = 1.10) were sure whether their educational curricula specifically addressed innovation and creativity competencies (Figure 3A). Staff agreed that their universities do not stimulate students to take part in interdisciplinary learning (Figure 3B). With respect to criteria for screening students prior to admission on such a course, staff were also divided in their opinion on whether additional assessment of prior knowledge is necessary before allowing access to an interdisciplinary module (see Figure 3C). It was generally accepted that additional assessment of personality is not needed before acceptance into an interdisciplinary module (84.9% ‘No’).

### Content

**Figure 4:**
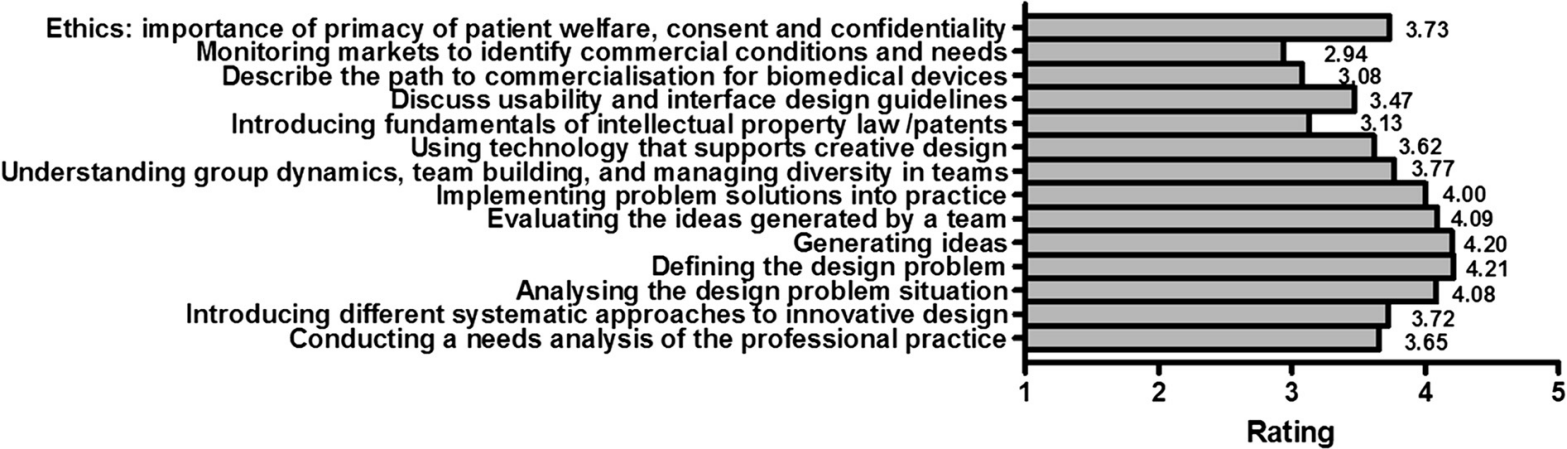
Mean ratings for staff and students’ preferred topics in course content – ratings on a scale of 1–5 from not important to most important.

Strong support was reported for classical issues/topics in creative design such as ‘Analysing the design problem situation’, ‘Defining the design problem’, ‘Generating ideas’, ‘Evaluating the ideas’, and ‘Implementing problem solutions into practice’ (see Figure 4).

Topics which received lesser scores included: ‘Understanding group dynamics and the management of diversity in teams’, ‘Introducing different systematic approaches to innovative design’, ‘Conducting a needs analysis of the professional practice’ and ‘Ethics: the importance of the primacy of patient welfare, consent and confidentiality’.

Those topics which scored least included: ‘Introducing fundamentals of intellectual property law and patents with application to biomedical devices’, ‘Discuss usability and interface design guidelines’, ‘Describe the path to commercialization for biomedical devices’, ‘Monitoring markets to identify commercial conditions and needs’.

### Instructional design

The following instructional approaches received *strong* support from both staff and students: ‘Be confronted with real-life design situations’; ‘Take on roles as found in real-life problem situations’; ‘Be given time to reflect on and discuss resources and examples’; ‘Perform tasks commissioned by real practitioners’; **‘**Be provided with examples’; ‘Applying a systematic approach to creative design’. The students indicated a strong preference for the following approaches: ‘Have teachers that are critical of the content they teach’; ‘Learn from professionals in my field’; ‘Have easy access to professionals in my field’. They were less appreciative of **‘**Have easy access to professionals in other fields’ and ‘Present the results of their work to practitioners in the target field’. Staff (but not students) indicated a high preference for ‘Work in interdisciplinary groups’.

### Assessment

**Figure 5:**
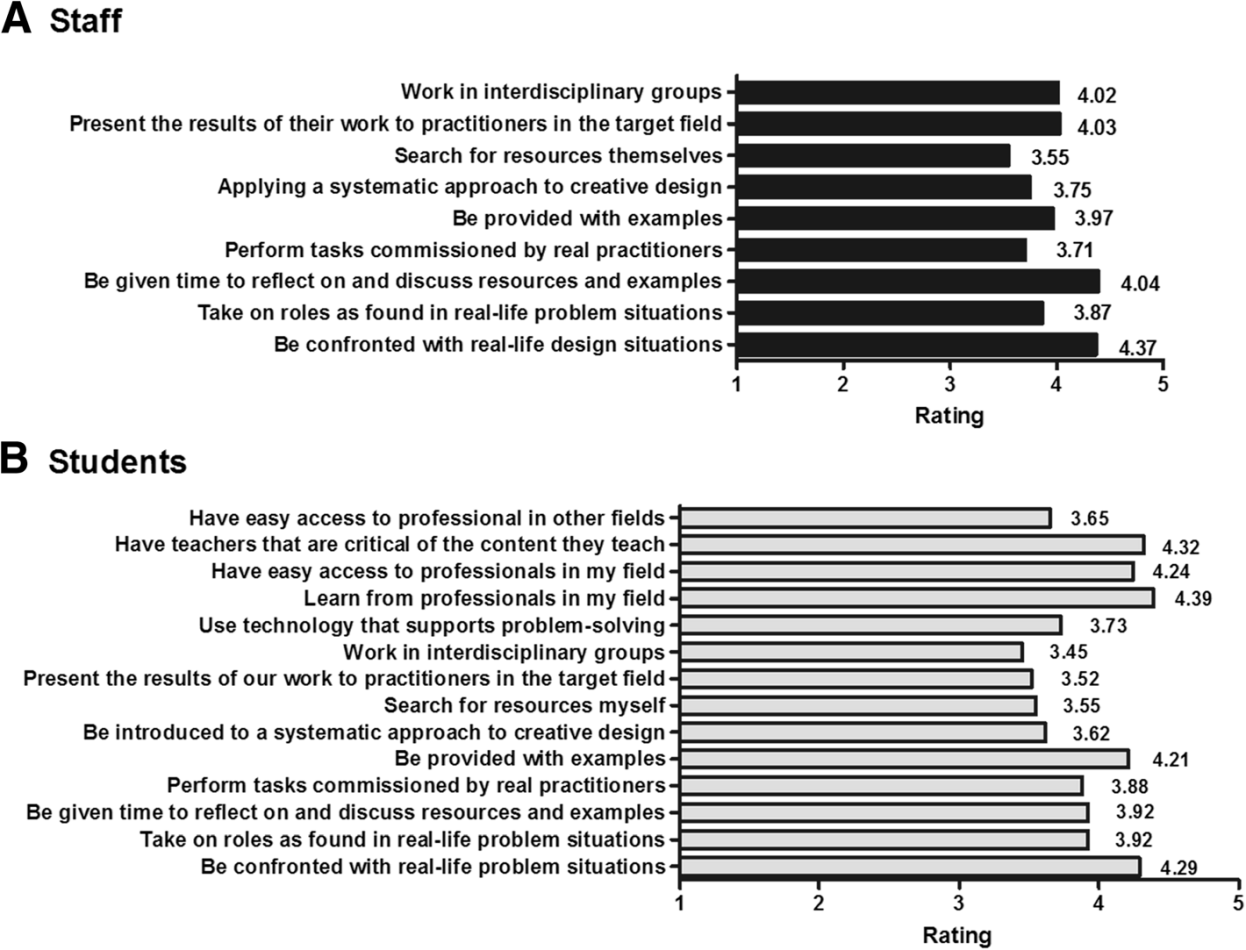
Mean ratings for staff [A] and students’ [B] preferred instructional approaches – ratings on a scale of 1–5 from not important to most important.

Staff ratings (M = 3.91, SD = 0.89) were higher than those of students (M = 3.30, SD = 1.17) on the item ‘Assessing creativity and innovation skills should use a variety of assessment methods (i.e. self-assessment, peer-assessment, e-portfolios, innovative assignments) rather than traditional assessments (i.e. formal tests, exams)’ (Figure 5A). Staff and student preferences for assessment methods for creativity were notable by their opposite reactions to the statement ‘Traditional assessment methods, such as formal tests and exams, can be used’ (M _staff_ = 3.13, SD = 1.04; M _students_ = 2.88, SD = 1.04; Figure 5B). Staff (M = 3.97, SD = 0.82) and students (M = 3.62, SD = 0.88) indicated a preference for teachers from contributing disciplines to design assessments, and that assessment design should result from a collaboration between staff and students (Figure 5C). Staff and students (M _staff_ = 3.66, SD = 1.07; M _students_ = 3.68, SD = 1.07) agreed that the focus of assessment should be on helping students to better understand how to handle a task (i.e. formative evaluation) rather than on formal judgment of students’ achievements (i.e. summative evaluation). Assigning marks in a course on creativity and innovation was perceived as a problem by staff (M = 3.38, SD = 1.06), but less so by students (M = 3.66, SD = 0.99).

### Engineering *vs*. medical student differences in current knowledge and attitudes towards interdisciplinary learning and teaching

Compared to medical students, engineering students were significantly *more likely* to agree or strongly agree with the following statements: ‘Interdisciplinary learning is indispensable for my professional development’ [U = 42714, z =4.19, *p* < 0.0001]; ‘Learning in interdisciplinary teams is to be preferred over learning with students from my own group’ [U = 43284, z =3.98, *p* < 0.0001]; ‘I consider all my fellow students to be creative’ [*χ*^2^ = 16.73, *p* < 0.0001].

When asked to rate a number of statements regarding organisational aspects of interdisciplinary learning, engineering students were more likely to agree or strongly agree that ‘My educational curriculum specifically addresses innovation and creativity competences’ [U = 20255, z = 13.91, *p* < 0.0001]; ‘My institution offers me incentives to engage in cross-disciplinary learning activities’ [U = 37996, z =6.227, *p* < 0.0001]; ‘The administrative system of my institution allows me to attend courses from other disciplines as part of my official curriculum’ [U = 33664, z =8.01, *p* < 0.0001].

When asked to specify learning preferences, engineering students were significantly *more likely* than medical students to favour: ‘Work in interdisciplinary groups’ [U = 40594, z =3.50, *p* < 0.0001]; ‘Work in team formations that aim at fostering innovation and creativity’ [U = 36188, z =5.56, *p* < 0.0001]; ‘Be given time to reflect on and discuss resources and examples’ [U = 41224, z =3.32, *p* = 0.001]; ‘Be introduced to a systematic approach to creative design’ [U = 38907, z =4.33, *p* < 0.0001]; ‘Evaluate the results from a systematic approach to creative design’ [U = 36972, z =5.24, *p* < 0.0001]; ‘Present the results of our work to practitioners in the target field’ [U = 40248, z =3.63, *p* < 0.0001]; ‘Use technology that supports problem solving’ [U = 37314.5, z =5.11, *p* < 0.0001].

Medical student ratings of the following were greater than those of engineering students: ‘Perform tasks commissioned by real practitioners’ [U = 43166, z =2.38, *p* = 0.017]; ‘Learn from professionals in my field’ [U = 42192.5, z =2.91, *p* = 0.004]; ‘Have easy access to professionals in my field’ [U = 39769.5, z =4.02, *p* < 0.0001].

Compared with medical students, engineering students reported greater ratings for assessments using a variety of assessment methods (i.e. self-assessment, peer-assessment, (e)-portfolios, innovative assignments) rather than traditional assessments (i.e. formal tests, exams) [U = 40261, z =3.58, *p* < 0.0001]. Medical students were more likely to respond that staff and students should design assessments collaboratively [U = 41689, z =2.95, *p* < 0.0001].

### Engineering *vs*. medical staff differences in current knowledge and attitudes towards interdisciplinary learning and teaching

Medical staff were *more* likely than engineering counterparts to agree that interdisciplinary learning is indispensable for professional development [U = 2000, z = 2.98, *p* < 0.01] and that the two groups of students use different vocabularies [U = 2165, z = 2.34, *p* < 0.05]. Medical staff were more likely to state that their existing curriculum did not address creativity and innovation [U = 1579, z = 4.53, *p* < 0.0001]. With respect to teaching preferences, medical staff indicated a stronger preference for ‘Performing tasks commissioned by real practitioners’ [U = 2196, z = 2.19, *p* < 0.05] and for ‘Use of role models’ [U = 2072, z = 2.63, *p* < 0.01]. Although engineering staff reported higher ratings for classical biomedical design content [‘Analyzing the design problem situation’, U = 2059, z = 2.74, *p* < 0.01], medical staff provided higher ratings for course content related to patient ethics, and content related to commercialization of outputs [‘Ethics: the important of the primacy of patient welfare, consent and confidentiality’, U = 1968, z = 3.01, *p* < 0.01; ‘Introducing fundamentals of intellectual property and patent with application to biomedical devices’, U = 2045, z = 2.71, *p* < 0.01]. Compared with their engineering counterparts, medical staff more strongly favoured the use of a variety of different assessment methods [U = 2096, z = 2.60, *p* < 0.01] in collaboration with students [U = 1889, z = 3.31, *p* < 0.001].

## Discussion

The aim of this study was to systematically examine knowledge and practice in interdisciplinary learning and teaching in engineering and medical schools in Ireland and Belgium (one institution in each country). The intention was to make available information which could inform the development of modules which support *interdisciplinary learning* of *interdisciplinary* design skills. We identified important differences both between disciplines and between staff and students.

### Attitudes towards interdisciplinary learning

The survey reveals an important educational need which is currently unmet. Although staff of both disciplines (but particularly medical staff) perceive interdisciplinary learning as useful for the professional development of students, both staff and students agreed that opportunities for academic interaction between the disciplines are limited. The external validity of this finding depends on how representative the institutions and samples studied are of a wider educational community. We have not formally evaluated this. Increasingly, accrediting organizations in the field of engineering and technology (e.g. IET, Institution of Engineering and Technology, ABET, Accreditation Board for Engineering and Technology) have included the ability to work in multidisciplinary teams [15], as well as the ability to apply quantitative methods within a multidisciplinary setting [16], among their program educational objectives. In the field of medicine, the importance of interdisciplinary team learning opportunities are now considered integral to medical education curricula e.g. [17].

Unlike staff and engineering students, medical students are not positively inclined towards interdisciplinary learning; they do not favour being grouped with learners from other disciplines. The majority of participants claim that engineering and medical students rarely interact and use different vocabularies; medical staff place greater emphasis on this as an obstacle to learning. The difference in terms of attitudes between engineering and medical student groups seems to justify a differentiated approach to preparations for participation in an interdisciplinary course. This would entail an examination of the relative motivational factors and priorities of students of medicine and engineering.

Although medical staff perceive interdisciplinary learning opportunities as important for their students, this perception is not shared by the student body. This is a potential barrier to the success of interdisciplinary initiatives. It is well established that undergraduates have prior perceptions/attitudes to interprofessional education and collaborative working. These are shaped by a complex mix of factors, including age, prior work experience and gender (with older students, females, and those with a positive interdisciplinary work experience being more positively inclined) [18]. Motivation to participate may contribute to success; however, the success of interprofessional learning is not necessarily influenced by whether students volunteer or are compelled to participate [19]. We were somewhat disappointed to note the relative reluctance of medical students to engage in interprofessional activities, but this has been noted previously [20]. If one assumes, as we do, that an interdisciplinary approach to innovation in healthcare is desirable, then it will be necessary to promote the benefits of such an approach in medical curricula and to and make opportunities to participate available. Identity formation may be more strongly expressed in medical students as compared with engineering students; if so, they may gravitate towards those with whom they share a professional identity. It is noteworthy, however, that medical staff clearly perceive the value of convergence and collaboration. This is important in that these are the likely promoters and providers of the opportunities mentioned above.

Few students and staff believe that everyone can be creative. This belief is not in line with recent research on creativity [21], where recent conceptualizations distinguish between level and style type of cognitive constructs [22]. Level types of construct refer to, for example, intelligence, knowledge and skills. A relevant question for identifying level constructs is “how much intelligence, knowledge, skills does the subject have? ‘In order to measure the style of creativity, we should also ask the question ‘in what way?’: People could be on the same cognitive level, but apply different creative styles, or have the same style but may operate on different levels. As a rule, everybody can be creative but s/he expresses his/her creativity in a different way (style), as creativity styles can be typified. Creativity methods, techniques and approaches can improve the quality of creative products – a finding in this study (see the item about being able to learn to be creative) that is in line with research on creativity [21,23]. Presentation of these ideas and evidence could form part of an introduction to innovation for engineering and medical students, thereby serving to both inform and motivate (particularly the latter).

### Instructional methods

The instructional approaches that receive most support from both teachers and students are the following: ‘Be confronted with real-life design situations’, ‘Take on roles as found in real-life problem situations’, ‘Be given time to reflect on and discuss resources and examples’; ‘Perform tasks commissioned by real practitioners’, **‘**Be provided with examples’. The value placed on authenticity, common to staff and students, is a well-recognized feature of successful inter-professional learning [20].

All students expressed a preference for having teaching staff who are critical of the content they teach, and having access to professionals and learning from professionals in their own field. This finding is in line with, for example, the cognitive apprenticeship approach [20,24].

### Interdisciplinary module content

The content related topics addressed in the BioApp module can be categorised based on the degree of support reported in our survey:

1) Classical creativity and innovation topics, such as ‘Analysing the design problem situation’, ‘Defining the design problem’, ‘Generating ideas’, ‘Evaluating the ideas’, and ‘Implementing problem solutions into practice’. These are the most oft-cited and well-characterised stages of creative problem solving design [25]. These topics received the strongest support from engineering staff, who understandably assigned greater importance than medical staff who may be less familiar with these approaches.

2) A second category of topics received less support from both staff and students. These included ‘Introducing different systematic approaches to innovative design’, ‘Conducting a needs analysis of the professional practice’, ‘Understanding group dynamics and the management of diversity in teams’, and ‘Ethics: the importance of the primacy of patient welfare, consent and confidentiality’. It was expected (and borne out) that staff and students from both backgrounds would recognize the importance of team dynamic, but that patient-specific issues would be more of a priority for medical respondents.

3) A third category of topics received least support. These included ‘Introducing fundamentals of intellectual property law and patents with application to biomedical devices’, ‘Discuss usability and interface design guidelines’, ‘Describe the path to commercialization for biomedical devices’, and ‘Monitoring markets to identify commercial conditions and needs’. Perhaps the lesser support reported is because these topics (with the possible exception of usability and interface issues) are not traditionally linked to creativity and innovation. We would argue that inclusion of these elements of content is justified because they form part of day-to-day experience and practice of engineering staff and students. Furthermore, marketing and intellectual property-related topics make the concept of interdisciplinary learning richer by including other professional fields than engineering and medicine. Interestingly this is recognized by medical staff, whose ratings of these topics were greater than those of their engineering counterparts. Our findings are also consistent with those from a survey of engineering graduates’ perception of their’industry-preparedness’, which revealed that a limiting step for engineering graduates has been the difficulty in understanding design constraints for other disciplines, accepting that patient-oriented factors can have a large impact on the acceptance, and integration of the technology into clinical practice [26].

Our findings suggest that medical staff and students are (appropriately and not surprisingly) focused on the clinical outcome of a design. They appreciate the importance of the above as a means to a clinical end. Although engineers are more open to interprofessional learning they may be less aware of the necessity that the design outcome meet a clinical need.

## Conclusion

We believe that convergent interprofessional undergraduate educational opportunities and modules are desirable. However, our data indicate that the design of such modules will need to take account of staff and student attitudes towards creativity and interdisciplinary learning. In the case of medical students, preparatory work may be useful. This could include outlining the relevance of each of the stages of the design process and promoting the benefits of interprofessional learning in general. In the case of engineering students, collaboration will expose them to clinical professionals and authentic clinical scenarios. These study conclusions are hedged with caveats (limitations of sample size due to online survey response rate, undetermined generalisability of the results), which might be addressed, at least partially, by expanding the study to other HEI institutions across the EU and, indeed, further afield. However, these data should serve to emphasise the constraints which apply to the introduction of novel solutions to the healthcare environment, the so-called ‘implementation gap’. Module design will need to address the dynamics of the interdisciplinary team because of the differences we have identified between medical and engineering students in their desire to use systematic approaches to learning.

## Competing interests

The authors declare that they have no competing interest.

## Authors’ contributions

HS, SS, CO’T, DB, CS, HD, KV, SVH, JMcS, GS, SO’F, PCM, and LB collectively conceived of the study, participated in its design and coordination, and helped to draft the manuscript. HS and SVH also collected the data, and CO’T performed the statistical analysis. All authors read and approved the final manuscript.

## Pre-publication history

The pre-publication history for this paper can be accessed here:

http://www.biomedcentral.com/1472-6920/14/14/prepub
